# Supplementary material for: Effect of the Marine Exercise Retreat Program on Thyroid-Related Hormones in Middle-Aged Euthyroid Women
Source: Int J Environ Res Public Health. 2023 Jan 14;20(2):1542. doi: 10.3390/ijerph20021542 (PMC9863542; doi:10.3390/ijerph20021542)
Supplement: Supplementary file 1 [file ijerph-20-01542-s001.zip › ijerph-2107507-supplementary.pdf]

Supplementary Materials:

Table S1. Correlation between fT4 level and HRV parameters in the fT4 group.

Variables	Total		Low		High	
	Correlation	*P*-value	Correlation	*P*-value	Correlation	*P*-value
Time Domain						
Mean HR	0.00165	0.99	-0.0798	0.670	0.182	0.32
SDNN	0.131	0.31	0.255	0.166	0.189	0.31
RMSSD	-0.0181	0.89	0.225	0.22	0.0731	0.70
Frequency Domain						
Total Power	0.112	0.39	0.326	0.07	0.102	0.58
LF power	0.122	0.35	0.282	0.12	0.0770	0.68
HF power	-0.102	0.43	0.219	0.24	-0.0503	0.79
Normalized LF	0.203	0.11	0.0898	0.63	0.0713	0.70
Normalized HF	-0.203	0.11	-0.0898	0.63	-0.0714	0.70
LF/HF Ratio	0.168	0.20	0.0189	0.92	-0.112	0.56

fT4, free thyroxine; HRV, heart rate variability; HR, heart rate; SDNN; standard deviation of NN intervals; RMSSD, root mean square of successive NN interval differences; LF, low frequency; HF, high frequency.

Table S2. Correlation between changes in TSH and changes in physical body measurement of participants before and after the marine exercise retreat program.

Variables	Total		Low		High	
	Correlation	*P*-value	Correlation	*P*-value	Correlation	*P*-value
ΔBMI	-0.0231	0.85	-0.134	0.46	-0.286	0.113
ΔBody Fat %	0.0910	0.47	-0.118	0.51	-0.118	0.51
ΔExercise Stress Score	0.0430	0.74	0.0174	0.92	0.0174	0.9
Blood Pressure						
ΔSBP	-0.0562	0.66	0.160	0.37	-0.25	0.17
ΔDBP	-0.053	0.67	0.193	0.28	-0.31	0.08
Thyroid-related hormones						
ΔTSH						
ΔfT4	0.153	0.22	0.23	0.19	0.055	0.76

Table S3. Correlation between changes in TSH and changes in HRV of participants before and after the marine exercise retreat program.

Variables	Total		Low		High	
	Correlation	*P*-value	Correlation	*P*-value	Correlation	*P*-value
Time Domain						
ΔMeanHR	-0.0292	0.82	0.202	0.28	-0.25	0.17
ΔSDNN	0.0453	0.73	-0.164	0.38	0.229	0.22

ΔRMSSD	-0.0549	0.67	-0.246	0.18	0.218	0.24
Frequency Domain						
ΔTotal Power	0.128	0.32	-0.153	0.41	0.305	0.09
ΔLF power	0.0387	0.77	-0.192	0.30	0.0937	0.62
ΔHF power	-0.119	0.36	-0.270	0.14	0.091	0.63
ΔNormalized LF	0.356	<0.01	0.209	0.26	0.372	0.04
ΔLF/HF Ratio	0.274	0.03	0.120	0.53	0.380	0.03

Table S4. Correlation between changes in fT4 and characteristics of participants before and after the marine exercise retreat program.

Variables	Total		Low		High	
	**Correlation**	***P*-value**	**Correlation**	***P*-value**	**Correlation**	***P*-value**
ΔBMI	-0.113	0.37	-0.125	0.49	-0.0721	0.69
ΔBody Fat %	0.192	0.12	0.460	<0.01	-0.00813	0.96
ΔExercise Stress	0.0188	0.88	0.264	0.14	-0.418	0.02
Blood Pressure						
ΔSBP	-0.120	0.34	-0.116	0.52	-0.0124	0.95
ΔDBP	-0.309	0.01	-0.343	0.05	-0.316	0.08
Thyroid-related hormones						
ΔTSH	0.153	0.22	0.0426	0.81	0.235	0.19
ΔfT4						

Table S5. Correlation between changes in fT4 and changes in HRV of participants before and after the marine exercise retreat program.

Variables	Total		Low		High	
	**Correlation**	***P*-value**	**Correlation**	***P*-value**	**Correlation**	***P*-value**
Time Domain						
ΔMean HR	-0.0301	0.82	-0.14	0.45	-0.121	0.51
ΔSDNN	-0.044	0.73	-0.00291	0.98	0.0419	0.82
ΔRMSSD	-0.086	0.51	-0.0625	0.73	0.152	0.41
Frequency Domain						
ΔTotal Power	-0.122	0.34	-0.0388	0.83	-0.0973	0.60
ΔLF power	-0.0707	0.58	-0.012	0.94	-0.142	0.44
ΔHF power	-0.0927	0.47	-0.198	0.28	0.2	0.28
ΔNormalized LF	0.098	0.44	0.0258	0.89	-0.168	0.36
ΔNormalized HF	-0.098	0.44	-0.0258	0.89	0.168	0.36
ΔLF/HF Ratio	0.12	0.36	-0.0302	0.87	0.0808	0.67
